# Essay on Tobacco

**Published:** 1841-04

**Authors:** 


Art. VIII.—*Essay on Tobacco.* By Henry Wilson Cleland, m.d., Lecturer on Medical Jurisprudence, &c.—*Glasgow*, 1840. 4to, pp. 68.

There is a class of persons, generally composed of old women of either sex, who have a mania for collecting: some delight in autographs; some in original poetry; some ride their hobby upon coins or crooked sixpences; and the author of "Ten Thousand a Year" tells us, that Mr. Quirk luxuriated in the gathering of locks of hair from the heads of executed or transported criminals. In like manner, Dr. Cleland appears to be "a collector and gatherer of other men's stuff;" but his monomania is tobacco; and, in his work, he seems to have strung together from the pages of his album, all that has been sung or said in praise or dispraise of it, and this, unfortunately, with little deduction or generalization of facts. The work, however, has afforded us an agreeable half-hour's recreation, and impressed us with a very favorable opinion of the author's talent for indefatigable research—a talent which we are quite sure he could profitably employ on less airy matters.

